# Plastid genetic engineering in *Solanaceae*

**DOI:** 10.1007/s00709-012-0391-9

**Published:** 2012-03-07

**Authors:** Jelli Venkatesh, Se Won Park

**Affiliations:** Department of Molecular Biotechnology, Konkuk University, 1 Hwayang-dong, Gwangjin-gu, Seoul, 143-701 Republic of Korea

**Keywords:** Genetic engineering, Homoplasmic, Plastid, *Solanaceae*, Transplastome

## Abstract

Plastid genetic engineering has come of age, becoming today an attractive alternative approach for the expression of foreign genes, as it offers several advantages over nuclear transformants. Significant progress has been made in plastid genetic engineering in tobacco and other *Solanaceae* plants, through the use of improved regeneration procedures and transformation vectors with efficient promoters and untranslated regions. Many genes encoding for industrially important proteins and vaccines, as well as genes conferring important agronomic traits, have been stably integrated and expressed in the plastid genome. Despite these advances, it remains a challenge to achieve marked levels of plastid transgene expression in non-green tissues. In this review, we summarize the basic requirements of plastid genetic engineering and discuss the current status, limitations, and the potential of plastid transformation for expanding future studies relating to *Solanaceae* plants.

## Introduction

The family *Solanaceae* consists of a number of economically important plants, including several major food crops, such as tomato, potato, eggplant, and pepper; ornamental crops, such as petunia; and medicinal crops, such as *Withania*, *Datura*, *Mandragora*, *Atropa*, and *Physalis*. The production of *Solanaceae* crops is constrained by several biotic and abiotic factors. Consequently, genetic engineering of *Solanaceae* plants has turned into a tool for the improvement of genotypes with increased tolerance to abiotic stresses, pests, and diseases. Some of the *Solanaceae* crop plants have become the targets of biofortification programs. Additionally, these crops have become bioreactors for the production of novel compounds, biopolymers, and pharmaceuticals (Van Beilen 2008; Huhns et al. 2008; Bock and Warzecha 2010; Bornke and Broer 2010; Petersen and Bock 2011). As nuclear transformation methods appear to be challenging in accomplishing some of these requirements, targeting the plastid genome becomes the most attractive alternative method. Plastids are plant cell organelles with many essential biosynthetic processes and pathways, such as photosynthesis, photorespiration, as well as metabolism of amino acids, lipids, starch, carotenoids, and other isoprenoids. Depending upon the organ type and environmental conditions, proplastids differentiate into a variety of plastids, such as chloroplasts in photosynthetic tissues, amyloplasts in storage tissue, and chromoplasts in fruits and flowers. Other specialized plastid types include gerontoplasts, the plastids of senescent leaves, which are important for resource allocation, oleoplasts, which are oil storage plastids, and etioplasts, which are found in the final stage of proplastid development in photosynthetic tissues in the dark (Hibberd et al. 1998; van Wijk and Baginsky 2011). Plastids have their own genome and protein-synthesizing machinery; however, nuclear genes encode most of the proteins used in plastids (Pogson and Albrecht 2011).

Plastid genetic engineering is a milestone approach for crop improvement programs, as plastid genomes can be effectively manipulated to attain desirable quality traits. Since plastids are maternally inherited in most of the crop species, the introduction of foreign genes into the plastid genome prevents pollen-mediated outcrossing (Bock 2001; Bock and Khan 2004; Maliga 2004) and also offers the possibility of polycistronic operon expression, thus enabling the stacking of multiple-expressed genes in a single transformed event (Staub and Maliga 1995). Furthermore, the polyploidy of the plastome in cells facilitates the high-level transgene expression (Maliga and Bock 2011). The expression of transgenes in transplastomic plants is more stable and uniform, as transgene integration always occurs by homologous recombination and is not affected by position effects or epigenetic gene-silencing mechanisms (Svab et al. 1990; Bock 2001), which occasionally occur in nuclear transformants (Kooter et al. 1999).

Daniell and McFadden (1987) provided the first proof of the uptake and expression of foreign genes in isolated plastids from dark-grown cucumber cotyledons. Soon after, Boynton et al. (1988) used high-velocity tungsten microprojectiles for plastid transformation of the unicellular alga *Chlamydomonas reinhardtii*. Since then, this concept has been extended to a number of crop species. In the *Solanaceae* family, chloroplast transformation has been reported in tobacco (Svab et al. 1990; O’Neill et al. 1993; Svab and Maliga 1993; Koop et al. 1996), tomato (Ruf et al. 2001; Nugent et al. 2005; Wurbs et al. 2007), petunia (Zubko et al. 2004), potato (Sidorov et al. 1999; Nguyen et al. 2005; Segretin et al. 2012; Valkov et al. 2011), and eggplant (Singh et al. 2010). Among these, tobacco has been the most important model crop for plastid genetic engineering, and a number of pharmaceutical and agronomically important genes have already been introduced into the tobacco plastid genome. However, its efficiency and applicability are rather limited, and reports of successful transgene expression are still scanty in other *Solanaceae* species. In this review, we summarize the various aspects of plastid transformation, including integration and expression of foreign genes into the plastid genome of important *Solanaceae* crops for various agronomical, industrial, and pharmaceutical applications. Furthermore, the current status and future prospects of plastid transformation in *Solanaceae* crop plants are also discussed in detail.

## Requirements for plastid transformation

### Plant regeneration system

For any successful study of genetic transformation, an efficient plant regeneration system is a prerequisite. The ability of *Solanaceae* plants to respond well in tissue culture, particularly plant regeneration from cultured seedling explants (cotyledons and hypocotyls), cells and protoplasts, has allowed the application of various biotechnology techniques for management of genetic resources and improvement of these crop plants. However, compared with tobacco regeneration systems, other *Solanaceae* family crops, such as tomato, potato, eggplant, and petunia regeneration systems, are several times lower, and significant differences have been observed in plastid transformation frequencies (Sidorov et al. 1999; Zubko et al. 2004; Gargano et al. 2005; Singh et al. 2010).

Sidorov et al. (1999) described the stable chloroplast transformation of potato by microprojectile bombardment of leaf explants; on average, one transplastomic event was recovered from 15 to 30 bombarded plates. Similarly, a low-transformation frequency of two per 21 bombarded plates was reported in eggplant with stem explants (Singh et al. 2010). The efficiency of plastid transformation in leaf explants of *Petunia hybrida* (Zubko et al. 2004) is of one plant per 10 bombarded plates. This was much lower than the frequency of one to five plants per bombardment obtained in *Nicotiana tabacum* (Svab and Maliga 1993; Iamtham and Day 2000), but it was comparable to plastid transformation efficiencies obtained in leaf explants of tomato (Ruf et al. 2001) and potato (Sidorov et al. 1999; Nguyen et al. 2005). However, with the improved regeneration protocol, transplastomic tomato lines at a higher frequency (on average, one to two transformants per bombardment) were obtained (Wurbs et al. 2007). Valkov et al. (2011) were able to regenerate about one shoot for every bombardment of potato leaf explants, and this efficiency corresponds to a 15–18-fold improvement, compared with previous reports.

### Gene transfer methods

The biolistic (gene gun) technique is the most widely used method, which has proven successful for delivering DNA into plastids in a variety of plant species. The disadvantages of this approach include the possibility of mechanical shearing of large plasmids during particle preparation or delivery, and a chemical reaction with tungsten (a reactive transition metal), which can promote the cleavage or modifications of DNA (Sanford et al. 1993). Furthermore, there is a possibility of occasional unintended co-transformation of chloroplasts and the nucleus (Elghabi et al. 2011a). However, the relatively high efficiency and technical simplicity make biolistic method, the most widespread technology for plastid transformation. The stable introduction of foreign DNA, via the polyethylene glycol (PEG)-mediated uptake of DNA by isolated chloroplasts, has also been conclusively demonstrated in tobacco and tomato (O’Neill et al. 1993; Koop et al. 1996; Eibl et al. 1999; Nugent et al. 2005). This method holds out the promise of the capacity to generate more cells with transformed plastids more readily than by the biolistic procedure. Moreover, the PEG method might be useful in species where plant regeneration is possible only from tissues containing plastids that are too small to tolerate the mechanical impact caused by microprojectiles (Koop et al. 1996). However, its feasibility is limited by high technical expertise, low shoot regeneration frequencies, chlorophyll deficiency (variegated leaves), and polyploidy in protoplast-derived plants (Meyer et al. 2009).

Knoblauch et al. (1999) demonstrated the direct microinjection of plasmid DNA into individual chloroplasts of the tobacco mesophyll cells with a galinstan expansion femtosyringe. Green fluorescent protein (*GFP*) gene driven by a chloroplast ribosomal RNA (rRNA) promoter was transiently expressed in attached leaves of tobacco after 24 h of injection and was subsequently detected in several chloroplasts in the injected cell. It was concluded that possibly plasmid DNA leakage occurred from the capillary or from the chloroplast on withdrawal of the capillary, which was subsequently taken up by other chloroplasts. Later, van Bel et al. (2001) explained it as a possible interplastidic GFP movement by transient connections known as stromules.

To exploit an alternative approach to the development of plastid transformation technology for other recalcitrant species of *Solanaceae*, Kuchuk et al. (2006) described the use of remote cytoplasmic hybrids, which was based on two somatic hybridization steps. Initially, they produced remote hybrids (cybrids) carrying the nucleus of tobacco, an easily transformable species, and plastids of the recalcitrant *Solanaceae* species. Cybrid protoplasts were then subjected to PEG-mediated plastid transformation (Koop et al. 1996). Later, protoplasts (with defective nucleus) from cybrid transplastomic plants were asymmetrically fused with protoplasts of the recalcitrant species, which originally provided the plastids in the first somatic hybridization step. Thus, the successful genetic transformation of plastids of five species of *Solanaceae*, such as *Scopolia carniolica*, *Physochlaina officinalis*, *Salpiglossis sinuata*, *Lycium barbarum*, and recombinant *N. tabacum/S. tuberosum* (potacco), was achieved through the use of high regeneration and transformation potential of intermediary “clipboard” hosts (plastid-defective *N. tabacum*). However, this approach has certain limitations, such as the number of steps involved, increased duration of genetic manipulations, genetic variability, and mitochondrial DNA recombination of cybrids. A similar approach was exploited by Sigeno et al. (2009) for intergeneric transfer of transformed chloroplasts from tobacco into petunia by asymmetric somatic hybridization. Tobacco strain whose plastids had been transformed with *aadA* and *MDAR* (monodehydroascorbate reductase) genes were used as a source of transformed plastids, and it was suggested that these studies could well expand the potential for the practical use of transplastomic tobacco and for the genetic improvement of other economically important *Solanaceae* crops. Moreover, cybridization between readily available transplastomic tobacco lines and cultivated *Solanaceae* crops would be simple with substantially reduced time duration.

### Plastid transformation vector and transgene expression

**Fig. 1:**
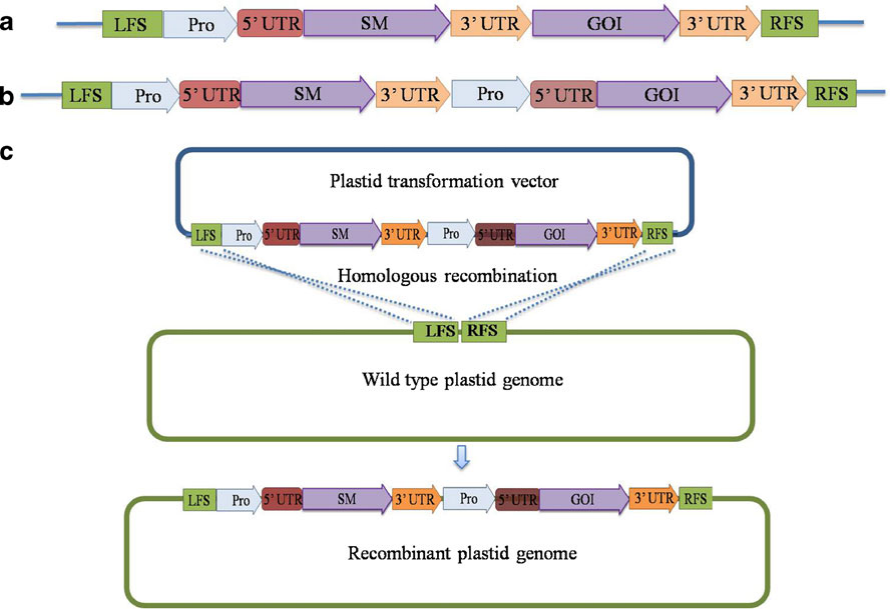
**a** Chloroplast expression vector cassette with *SM* and *GOI* driven by separate promoters. **b** Chloroplast expression vector cassette with *SM* and *GOI* driven by a single promoter (Pro). **c** Homologous recombination between plastid transformation vector and wild-type plastid genome

In general, two distinct components are required to construct the final chloroplast transformation vector system: a vector containing the flanking sequences, left flanking sequence, and right flanking sequence, and the sequences required for efficient transgene expression (expression cassette) (Fig. 1a, b). Flanking sequences are the DNA sequences from the chloroplast genome, which are homologous to the desired site of integration. Their function is to facilitate the site-specific recombination and define the integration site of the transgene. Therefore, the flanking sequence must be specific to the plastid genome being targeted; these sequences are approximately 1 kb in size and are located on either side of the expression cassette (Fig. 1a, b). The most commonly used site of integration is the transcriptionally active intergenic region between the *trnI–trnA* genes within the rRNA operon, which is placed in the inverted repeat (IR) region of the chloroplast genome (Verma and Daniell 2007). The expression cassette (Fig. 1) includes a selectable marker (*SM*) gene and the gene of interest (*GOI*), either driven by a single promoter (Pro) (Fig. 1a) or by separate promoters (Fig. 1b), flanked by the 5′ and 3′ untranslated regions (UTRs) of plastid genomes.

Earlier, plastid transformations were carried out with vectors designed for tobacco transformation, as the tobacco homologous flanking sequences present in these transformation vectors share a very high homology to the corresponding sequences of plastid DNA in other *Solanaceae* crops (Sidorov et al. 1999; Berger et al. 2005; Nguyen et al. 2005). Therefore, the efficient integration of such sequences in these species via homologous recombination was apparent. However, Ruhlman et al. (2010) emphasized the role of endogenous regulatory elements and flanking sequences for efficient chloroplast expression. Transplastomic tobacco or lettuce lines with heterologous *psbA* promoters, 5′ UTR and 3′ UTRs showed reductions of 80 % anthrax protective antigen (PA) and 97 % human proinsulin fused with the cholera toxin, B-subunit (CTB-Pins) expression, when compared with endogenous *psbA* regulatory elements. Thus, the use of heterologous gene regulatory elements could substantially reduce the transgene expression, due to transcript instability, differential affinity for RNA-binding proteins and reduced translational efficiency (Ruhlman et al. 2010).

Variations in accumulation of transgene transcripts and their differential translatability are attributed to the plastid constructs with different promoters and UTRs (Eibl et al. 1999; Zhou et al. 2008). For instance, a 3-fold increase in the amount of *uidA* transcripts was achieved with exchange of the *rpl32* 3′ UTR for the *rbcL* or *psbA* 3′ UTRs, but did not significantly influence the amount of β*-*glucuronidase (GUS) protein accumulation in transplastomic tobacco plants (Eibl et al. 1999). The *atpI* promoter and the 3′ UTR from the tobacco *rps16* gene facilitated expression of the bacterial lycopene β-cyclase gene, with 4-fold enhanced pro-vitamin A content of the tomato fruits (Wurbs et al. 2007). Similarly, a drastic increase in the abundance of HIV fusion antigen, *p24-nef*, messenger RNA (mRNA) was achieved in tomato fruit through controlling the transcription of transgenes using the full-length tobacco plastid rRNA operon promoter (Zhou et al. 2008).

Recently, Valkov et al. (2011) demonstrated the roles of alternative 5′ UTR and 3′ UTRs on transcript stability and translatability of plastid genes in potato. A significant positive effect of *clpP* 5′ regulatory sequences on translatability, particularly in non-green plastids was found (Valkov et al. 2011), which is in agreement with expression profile analyses that indicated *clpP* as being one of the less downregulated genes in tubers, compared with leaves (Valkov et al. 2009). In leaves, the accumulation of GFP was about 4 % of the TSP, with constructs containing the plastid rRNA operon promoter (*rrn*) and a synthetic *rbcL*-derived 5′ UTR, whereas, with the *clpP* promoter and *clpP* 5′ UTR sequence from the *clpP* gene, it was about 0.6 % of the TSP. However, in tubers, GFP expression was equally detectable (up to approximately 0.02 % of the TSP) with plants transformed with both constructs (Valkov et al. 2011). As protein accumulation in plants containing constructs with the *rrn* promoter is generally accompanied by high expression in leaves, a potential use of the *clpP 5*′ regulatory sequences can be envisaged in cases where recombinant protein accumulation is required in amyloplasts, but not in chloroplasts (Valkov et al. 2011). Apparent differences were also observed between the constructs with distinct 3′ UTRs, but the same 5′ regulatory sequences, suggesting the role of 3′ UTRs on transcript stability and accumulation in amyloplasts (Valkov et al. 2011). Plants with the bacterial-derived *rrnB* terminator accumulated five- and 7-fold more *GFP* transcripts than plants with *psbA* and *rpoA* 3′ UTRs, respectively, suggesting a positive role of the *rrnB* terminator in mRNA stability (Valkov et al. 2011). Segretin et al. (2012) used plastid constructs with flanking sequences and regulatory elements derived from tobacco and achieved a high-level expression of GUS protein (up to 41 % of the TSP) in mature transplastomic potato leaves, which was comparable to expression levels obtained in tobacco. Their results suggest that heterologous flanking sequences and regulatory elements derived from tobacco can also be effectively used for plastid transformation of other *Solanaceae* species.

### Selection system

Plastid genetic engineering in higher plants typically involves a stable introduction of the antibiotic-resistance gene as a selection marker, along with the gene of interest. For any successful plastid transformation, an efficient selection marker is required for organelle sorting out during repeated cell divisions in vitro, in order to achieve regeneration of homoplasmic transplastomic shoots (Bock 2001; Maliga 2004). Initially, the relatively low transformation frequency was observed with antibiotic-resistant 16S rRNA allele as a selectable marker. This was most probably due to the recessive mode of action of the rRNA marker during the selection phase as it conferred antibiotic resistance only to those few chloroplast ribosomes that had received their 16S rRNA molecule from hardly any initially transformed plastid DNA copies in a cell. Thus, antibiotic-resistant 16S rRNA allele was not considered as an efficient selectable marker for plastid transformation (Bock 2001; Nugent et al. 2005). However, vectors with naturally occurring recessive point mutations may be more acceptable than dominant bacterial antibiotic-resistance genes and may obviate the need for marker-excision technologies. Nevertheless, the point mutation conferring antibiotic insensitivity cannot be subsequently removed, it being a part of an essential plastid gene associated with the production of functional ribosomes (Nugent et al. 2005). In contrast, antibiotic-inactivating marker genes provide dominant drug resistance to the recipient chloroplast, and even a single transformed plastid genome copy is sufficient to detoxify the entire organelle (Bock 2001).

Plastid transformation is usually achieved with the use of antibiotic-resistance genes, such as *nptII*, *aphA-6*, and *aadA* genes. The foremost and commonly used chloroplast-specific antibiotic resistance marker is *aadA*, conferring resistance to a number of antibiotics of the aminoglycoside type, including spectinomycin and streptomycin (Goldschmidt-Clermont 1991). Transformation efficiency with the chimeric *aadA* gene is about 100-fold greater than the antibiotic resistance conferred by mutations in 16S rRNA genes (Svab et al. 1990; Svab and Maliga 1991). The most efficient and routinely used selectable markers have been spectinomycin and kanamycin selections; however, the kanamycin selection appears to be less efficient, as it produces a significant background of nuclear transformants (Svab and Maliga 1993). Recently, Li et al. (2011) used chloramphenicol acetyltransferase (CAT) as a selectable marker and obtained homoplastic tobacco chloroplast transformants with no spontaneous antibiotic-resistant mutants. On the basis of their results, they proposed that the *CAT* gene can be used as a novel selectable marker for plastid transformation in higher plants. On the contrary, use of the herbicide selection system is known to have a detrimental effect on the plant system. The bacterial bialaphos resistance (*bar*) gene, coding for phosphinothricin acetyltransferase (PAT), has been used as a plastid selection marker. The plastidial expressed *bar* gene would not be suitable for the direct selection of transplastomic lines due to the inefficient inactivation of phosphinothricin in the cytoplasm by the plastid-localized PAT even with the *bar* gene expressed at a higher level (>7 % of the TSP). This indicates that subcellular localization rather than the absolute amount of the enzyme is critical for direct selection of transgenic clones (Lutz et al. 2001).

Green fluorescent protein has been an excellent candidate for non-destructive monitoring of gene expression in subcellular compartments, such as chloroplasts, mitochondria, endoplasmic reticulum, actin cytoskeleton, and nuclei, through the addition of signal peptides (Koehler et al. 1997). GFP was transiently expressed in non-green tissues of a number of crops after biolistic bombardment (Hibberd et al. 1998). It has been reported that high expression of GFP could affect the plant morphology or inhibit plant regeneration (Haseloff and Siemering 1998). However, a high level expression of GFP (5 % of the TSP) in chloroplasts of potato had no apparent deleterious effect, perhaps due to the organelle compartmentalization (Sidorov et al. 1999), suggesting that GFP expression would be a valuable marker for screening of non-photosynthetic plastid transformants at the early stages of selection. Furthermore, several photosynthesis-deficient plastid mutants (*ΔpetA*, *Δycf3*, *ΔrpoA*, and *ΔrbcL*) have been used for the development of a phenotypic selection system (Klaus et al. 2003; Kode et al. 2006). The reconstitution of the deleted genes in transformants permits the regeneration of photoautotrophic-transformed shoots with a visually distinct phenotype comparable to the mutant phenotypes, and overcomes the problems associated with plastid transformation, such as the occurrence of spontaneous mutants or nuclear insertions. In addition to the benefits offered by the visual selection, they also facilitated the rapid recovery of homoplasmic lines. A combination of dominant selectable markers with a visual screening system for the early and conclusive detection of plastid transformants has also been successfully achieved (Klaus et al. 2003).

### Excision of marker genes and some alternative strategies

Incorporation of a selectable marker gene along with the gene of interest in the plastid genome is essential to obtain homogeneous plastid genome copies in a plant cell. However, once homoplasmic transplastomic plants are obtained, the marker gene is no longer necessary, and removal of the marker gene enables multiple cycles of transformation with the same selection marker gene. Moreover, integration of antibiotic/herbicide-resistance genes in transformed plants raises environmental and health concerns toward the commercialization of transgenic plants. Therefore, efficient methods for complete elimination of marker genes from plastid transformants are necessary to ensure the safety of human health and the environment.

To date, a number of strategies have been employed for the removal of marker genes from the transplastomic plants (reviewed in Lutz and Maliga 2007; Upadhyaya et al. 2010; Day and Goldschmidt-Clermont 2011). One of these approaches is based on the deletion of marker genes by spontaneous homologous recombination via direct repeats flanking the marker gene (Iamtham and Day 2000). However, homology-based marker excision relies on secondary recombination and segregation of plastid DNA in an inherently genetically unstable, heteroplastomic plant, which makes the attainment of marker-free transplastomic plants difficult (Lutz and Maliga 2007). The second approach relies on co-transformation–segregation of selectable and non-selectable marker genes in a genetically unstable, segregating plastid DNA population (Ye et al. 2003). Nevertheless, obtaining stable transplastomic plants through genetically unstable and segregating plastid genome populations is difficult. The third approach is the excision of marker genes with the use of site-specific phage recombination systems, Cre/*LoxP* or phiC31/*attB*/*attP* (Lutz et al. 2007). This system requires additional steps of integration and removal of plastid-targeted phage recombinases from transplastomic plants. Fourth approach is the transient cointegration of the marker gene, which relies on antibiotic/phenotypic selection of plastid mutants (Klaus et al. 2004). However, this method requires extra effort to isolate and propagate the plastid mutants needed to facilitate the identification of desirable recombination events (Day and Goldschmidt-Clermont 2011). Considering these limitations, the development of a more sophisticated system for generation of marker-free transplastomic plants becomes a necessity, which could facilitate the production of transplastomic plants with a minimum number of manipulations, thereby reducing the possibility of any unwanted recombination effects.

Alternative strategies that have also been reported, without the use of antibiotic/herbicide selection markers, are using those genes that are naturally present in plants and through conferring a metabolic or developmental advantage to the transformants. Several sugar or sugar alcohols and amino acid analogues have been used as positive selection systems for the production of marker-free transgenic plants (Penna et al. 2002; Barone et al. 2009). Attempts were made to use betaine aldehyde dehydrogenase (*BADH*) as a selection marker gene (Daniell et al. 2001a; Day and Goldschmidt-Clermont 2011), which accumulates in the plastids of a few plant species adapted to dry and saline environment, which is involved in the conversion of toxic betaine aldehyde (BA) to non-toxic glycine betaine (Rathinasabapathi et al. 1994). However, it was found that selection with BA was non-reproducible, and it can be stated that the BA selection method is not a reliable approach for plastid transformation (Maliga 2004; Whitney and Sharwood 2008). Barone et al. (2009) developed a selection system based on the tryptophan feedback-insensitive anthranilate synthase (AS) α-subunit gene of tobacco (*ASA2*) as a selective marker, with indole analogue 4-methylindole (4MI) or the tryptophan analogue 7-methyl-DL-tryptophan (7MT) as the selection agents. The use of the *ASA2*/7MT or 4MI selection system could facilitate the expansion of plastid transformation technology to crops that are naturally resistant to spectinomycin and for which a specific selection system still has to be established (Barone et al. 2009).

Features that have been the target of plastid genetic engineering of *Solanaceae* crop plants include increased photosynthetic efficiency, biofortification, abiotic stress tolerance, herbicide resistance, pest and disease resistance, and the use of plants as factories for producing biopolymers and biopharmaceuticals. In the following sections, the progress made in these areas will be discussed in detail.

## Photosynthetic efficiency

Chloroplast is an obvious candidate for increasing photosynthetic efficiency, providing one of the attractive avenues to increase the crop yields. One such example is the hybrid RuBisCO, which could lead to an increase in the production of food, fiber, and renewable energy (Spreitzer and Salvucci 2002; Genkov et al. 2010). Over the past few years, extensive work has been carried out to engineer RuBisCO to alter its enzymatic properties (Kanevski et al. 1999; Andrews and Whitney 2003; Raines 2006; Parry et al. 2007) and, in particular, its large chloroplast-encoded catalytic subunit as a target for engineering to increase the net CO_2_ fixation in photosynthesis. Naturally occurring RuBisCOs with superior catalytic turnover rates and better specificity have been found among the red algae and C_4_ plant species (Von Caemmerer and Evans 2010). Kanevski et al. (1999) demonstrated the feasibility of using a binary system in which different forms of the large subunit of RuBisCO gene (*rbcL*) are constructed in a bacterial host and then introduced into a vector for homologous recombination in transformed chloroplasts to produce an active, chimeric enzyme in vivo. Transplastomic tobacco lines expressing the sunflower *rbcL* gene synthesized a hybrid form of enzyme with large subunits of sunflower and small subunits (*rbcS*) of tobacco with enzymatic properties similar to the hybrid enzyme (Kanevski et al. 1999). However, transplastomic line expressing the cyanobacterial *rbcL* gene failed to assemble correctly using the tobacco chloroplast protein folding machinery, and it neither produced the large subunit nor showed any enzyme activity. Similarly, Zhang et al. (2011) produced transplastomic tobacco plants with the *rbcL* gene replaced by tomato-derived *rbcL* and demonstrated that the tomato large subunit was assembled with the tobacco small subunit into functional RuBisCO.

Although the large subunit of RuBisCO contains the catalytic active site, small subunit can also influence the carboxylation catalytic efficiency and CO_2_/O_2_ specificity of the enzyme, as well as contribute significantly to the overall catalytic performance of RuBisCO (Genkov et al. 2010; Whitney et al. 2011). However, engineering the native or foreign *rbcS* genes in higher plants remains an inexplicable challenge due to the multiple *rbcS* copies that are located in the nucleus, which essentially precludes *rbcS* from targeted mutagenic or replacement strategies (Whitney and Sharwood 2008). All these hybrid or foreign RuBisCO enzymes, even when enzymatically competent, displayed impaired biogenesis *in planta*, mainly due to the problems with subunit folding and assembly. Consequently, many of the resultant plants suffered severe defects in photosynthesis and growth. Thus, manipulation of endogenous RuBisCO has been largely unsuccessful in terms of improving enzyme activity (Zhang et al. 2011). Although the recent developments in improving the performance of RuBisCO seems to be reluctant, this research has provided novel insights into structural and functional relationships and has considerably enhanced our understanding of this key enzyme, providing new opportunities to develop more productive crop plants (Whitney and Andrews 2001; Zhang et al. 2002; Dhingra et al. 2004). Additionally, the engineering of metabolic and photosynthetic activities for increasing sink strength, especially in non-leaf sinks, such as fruits and tubers, will have a tremendous potential to improve the crop yield.

## Abiotic stress tolerance

Conventional plant breeding methods to accelerate the abiotic stress tolerance of *Solanaceae* crop plants have met with limited success, plus efforts to improve the abiotic stress tolerance are complicated by genetic complexity (Waterer et al. 2010). Therefore, genetic engineering would provide a potentially useful tool for improving abiotic stress tolerance of the *Solanaceae* crops with newly developed crop varieties to adhere to high yield and quality expectations.

Sigeno et al. (2009) developed the transplastomic petunia containing genetically transformed tobacco chloroplast, expressing monodehydroascorbate reductase (MDAR), one of the antioxidative enzymes involved in the detoxification of the ROS under various abiotic stresses. The *MDAR* gene was transcribed in the somatic cybrids of petunia as the transplastomic tobacco plants. Similarly, transplastomic tobacco plants expressing either a tobacco mitochondrial superoxide dismutase (*MnSOD*) or an *E. coli* glutathione reductase (*gor*) gene, which is associated with the scavenging of ROS showed improved tolerance for various abiotic stresses. Thus, the level of enzymes associated with ROS scavenging can be effectively modified through direct chloroplast transformation (Poage et al. 2011).

The possibility of altering the unsaturation levels of fatty acids in plant lipids by plastid genetic engineering could provide the plants with abiotic stress tolerance as well as improved nutritional value. Craig et al. (2008) produced transplastomic tobacco plants, which express a *Delta-9 desaturase* gene from either the wild potato species *Solanum commersonii*, or the cyanobacterium, *Anacystis nidulans*, which controls the insertion of double bonds in fatty acid chains, and demonstrated the increased cold tolerance in transplastomic plants with altered leaf fatty acid profiles. Earlier integration and expression of a *Delta-9* desaturase gene has also been demonstrated in potato plastids in order to achieve higher content of unsaturated fatty acids, a desirable trait for stress tolerance of higher plants, in addition to improved nutritional value (Gargano et al. 2003, 2005).

To cope with adverse environmental conditions, many plants express low molecular weight compounds collectively called osmoprotectants, which are typically sugars, alcohols, proline, and quaternary ammonium compounds (Glick and Pasternak 1998). Transplastomic tobacco plants, which express the yeast trehalose phosphate synthase (*TPS1*) gene, showed an accumulation of trehalose several times higher than the best surviving nuclear transgenic plants without any pleiotropic effects (Schiraldi et al. 2002; Lee et al. 2003). Another highly effective osmolyte glycine betaine (GB) is known to accumulate only in few plant species during drought or high salinity and protects the plant by maintaining an osmotic balance within the cell (Robinson and Jones 1986; Rathinasabapathi et al. 1994). Transplastomic tobacco plants, which were transformed with a gene encoding choline monooxygenase (BvCMO) from *Beta vulgaris*, were able to accumulate GB in leaves, roots, and seeds and showed improved tolerance to toxic levels of choline, in addition to exhibiting tolerance to salt/drought stress, when compared with wild-type plants. Transplastomic plants also demonstrated higher net photosynthetic rates and an increased quantum yield of photosynthesis, even in the presence of salt stress (Zhang et al. 2008).

## Herbicide resistance

Plastid genetic engineering provides increased containment of herbicide resistance genes as plastid genes are not transmitted by pollen. The most commonly used herbicide, glyphosate, is a broad-spectrum systemic herbicide known to inhibit the plant aromatic amino acid biosynthetic pathway by competitively inhibiting the 5-enolpyruvylshikimate-3-phosphate synthase (EPSPS), a nuclear-encoded chloroplast-targeted enzyme involved in the biosynthesis of aromatic amino acids (Bock 2007). Most of the transgenic plants resistant to glyphosate are typically engineered to overexpress the *EPSPS* gene (Ye et al. 2001). As the target of glyphosate resides within the chloroplast, chloroplast transgenic engineering is an ideal strategy for developing glyphosate resistance in plants. Transgenic tobacco plastids expressing the *EPSPS* gene resulted in the accumulation of over 250-fold, EPSPS enzymes, when compared with nuclear transgenics (Ye et al. 2001). However, such increased levels of glyphosate-resistant EPSPS did not correlate to increased tolerance to glyphosate. One reason for this discrepancy between protein level and tolerance was that the nuclear-encoded gene is expressed at a high enough level to confer resistance in the appropriate cell types, whereas the plastid transgene is not (Ye et al. 2001). Transplastomic tobacco plants expressing the bacterial *bar* gene linked with spectinomycin resistance (*aadA*) gene for selection of transformants showed a significantly elevated expression level of phosphinothricin acetyltransferase and exhibited field-level tolerance to Liberty, an herbicide containing PPT (Lutz et al. 2001).

On the contrary, tobacco plastome engineering of the *hppd* (4-hydroxyphenylpyruvate dioxygenase) gene from *Pseudomonas fluorescens*, which is part of the biosynthetic pathway leading to plastoquinone and vitamin E biosynthesis (Dufourmantel et al. 2007), resulted in the accumulation of HPPD to approximately 5 % of the TSP in transgenic chloroplasts with strong tolerance to the triketone herbicide, Isoxaflutole. Transplastomic tobacco seedlings overexpressing the barley *hppd* gene showed a higher resistance to another triketone herbicide, Sulcotrione (Falk et al. 2005). Similarly, Wurbs et al. (2007) produced the transplastomic tomato expressing bacterial lycopene ß-cyclase gene, resulting in increased levels of herbicide tolerance to 2-(4-chlorophenylthio)-triethylamine (CPTA), which specifically inhibits lycopene ß-cyclase activity.

## Pest and disease resistance

The *Solanaceae* family includes some of the most widely cultivated vegetable crops, which are susceptible to several pests and diseases (Afroz et al. 2011; Girhepuje and Shinde 2011). Pest and disease-resistant transgenic plants would provide an effective built-in pest and disease control, in addition to protecting the environment from adverse effects of agrochemicals. Despite significant progress in nuclear transformation, plastid engineering might be particularly useful in those cases where successful resistance engineering crucially depends on high expression levels of the resistance gene and increased gene containment and biosafety.

Most of the approaches make use of insecticidal protoxins produced by a variety of *Bacillus thuringiensis* strains for pest control. Expression of *Cry* genes in the plastid genome does not require adjustment of codon usage or any other sequence manipulations (McBride et al. 1995; De Cosa et al. 2001; Chakrabarti et al. 2006). Moreover, multiple gene stacking by polycistronic expression of transplastomic chloroplasts would avoid the retransformation and additional selectable marker gene integration in the plant genome by conventional nuclear gene pyramiding via *Agrobacterium-*mediated gene transfer (Meiyalaghan et al. 2010). In addition, the absence of insecticidal proteins in transgenic pollen eliminates toxicity to pollen-feeding non-target insects, thereby increasing the efficacy and safety of transgenic plants throughout the growing season (De Cosa et al. 2001). De Cosa et al. (2001) reported the high expression of insecticidal Bt-toxins in tobacco chloroplasts with no obvious phenotypic defects and shown to process a bacterial operon properly, which expressed the insecticidal Cry2Aa2 proteins at levels up to 46 % of the TSP (solubilized in NaOH, as crystalline Cry inclusion bodies are soluble at high alkaline pH). In contrast, significantly delayed plant development of transplastomic tobacco plants expressing *Cry9Aa2* gene was associated with an increased accumulation of the TSP (∼10 % in cellular fraction and ∼20 % in membrane fraction) (Chakrabarti et al. 2006). Therefore, the possibility of phenotypic defects in the transplastomic plants expressing these insecticidal proteins cannot be ruled out.

DeGray et al. (2001) first reported the disease-resistant transplastomic tobacco plants conferring resistance against a broad range of pathogens expressing MSI-99, an antimicrobial peptide (AMP), and an analog of maganin-2, a defense peptide secreted from the skin of the African clawed frog (*Xenopus laevis*). AMPs are helical antimicrobial peptide that confers protection against many prokaryotic organisms, due to its high specificity for negatively charged phospholipids, which are typically found in outer membranes of bacteria and fungi (Houston et al. 1997; Biggin and Sansom 1999). In vitro assays with leaf/protein extracts from transplastomic plants showed a highly significant inhibition of growth of bacterial pathogen *Pseudomonas syringae* pv. tabaci and pre-germinated spores of three fungal species, *Aspergillus flavus*, *Fusarium moniliforme*, and *Verticillium dahliae*. *In planta* assays with the bacterial pathogen, *P. syringae* pv. tabaci, and the fungal pathogen, *Colletotrichum destructivum*, showed areas of necrosis around the point of inoculation in control leaves, whereas transplastomic plant leaves showed no signs of necrosis, demonstrating a high-dose release of the peptide at the site of infection by chloroplast lysis (DeGray et al. 2001).

## Biofortification

One of the advantages of genetic engineering is the cost-effective production of nutritional compounds, which have the potential to improve the human nutrition and health status. The ability to express multiple genes as an operon makes chloroplast genetic engineering an attractive method for engineering the nutritionally important metabolic pathways. Carotenoids are essential pigments of the photosynthetic machinery in plants and an indispensable component of the human diet. In addition to being potent antioxidants, they also provide the vitamin A precursor, β-carotene (Apel and Bock 2009). The carotenoid biosynthetic pathway, localized in the plastid, has been thoroughly investigated and several strategies have been used to metabolically engineer the carotenoid biosynthesis in crop plants (Wurbs et al. 2007; Lopez et al. 2008; reviewed in Lu and Li 2008). Plastid engineering of *Solanaceae* crop plants for enhanced β-carotene synthesis would also be possible by overexpression of a single or combination of two or three bacterial genes, *CrtB*, *CrtI*, and *CrtY*, encoding phytoene synthase, phytoene desaturase, and lycopene β-cyclase, respectively.

Wurbs et al. (2007) demonstrated the feasibility of engineering nutritionally important biochemical pathways in non-green plastids by transformation of the chloroplast genome of tomato. The transplastomic tomato expressing bacterial lycopene β-cyclase gene resulted in the conversion of lycopene to β-carotene with 4-fold enhanced β-carotene content of the fruits. Similarly, Apel and Bock (2009) produced the transplastomic tomato fruits expressing the lycopene β-cyclase genes from the eubacterium, *Erwinia herbicola*, and the higher plant daffodil (*Narcissus pseudonarcissus*). Although expression of the bacterial lycopene β-cyclase did not strongly alter carotenoid composition, expression of the daffodil lycopene β-cyclase efficiently converted lycopene into provitamin-A (β-carotene), plus accumulated β-carotene with provitamin-A levels reaching 1 mg/g dry weight.

All the vitamin E synthesis enzymes are nuclear encoded and imported into the plastid, except for HPPD, which is active in the cytoplasm, catalyzing an early step (conversion of 4-hydroxyphenylpyruvate to homogentisate) in the biosynthetic pathway of tocopherols (Garcia et al. 1999). The *hppd* gene from *Hordeum vulgare*, which was expressed in tobacco plastids, accumulated more than twice as much α-tocopherol in transplastomic tobacco leaves than wild-type tobacco leaves (Falk et al. 2005). However, overexpression of the *hppd* gene in plastids did not prove to be advantageous. It has been suggested that homogentisate synthesized in the plastids is not directly accessible to the binding site of the homogentisate phytyltransferase/homogentisate solanesyltransferase, the next enzymes of the tocopherol and plastoquinone biosynthetic pathway (Falk et al. 2005). It was proposed that at least five genes in the vitamin E pathway have to be upregulated in order to enhance its accumulation significantly in oil seed crops (Kinney 2006). Likewise, transformation of the plant chloroplast with any or all these genes, via a multigene construct and a single promoter, would allow their expression to be several times higher (Bock 2007; Day and Goldschmidt-Clermont 2011).

Plastid engineering holds great promise for manipulation of fatty acid biosynthesis pathway genes and contributes to improved food quality and biofuel production. Several biosynthetic reactions of fatty acid biosynthesis are localized in the plastids, which can be effectively targeted via plastid engineering to increase fatty acid production in plants. A number of studies have demonstrated significant progress towards the goal of improving plant fatty acids (reviewed in Rogalski and Carrer 2011). Plastidic acetyl-CoA carboxylase (ACCase) is a key enzyme regulating the rate of de novo fatty acid biosynthesis in plants, composed of three nuclear-encoded subunits and one plastid-encoded accD subunit. Madoka et al. (2002) replaced the promoter of the *accD* operon in the tobacco plastid genome with a plastid rRNA-operon promoter (*rrn*), which directs enhanced expression in photosynthetic and non-photosynthetic organs, and successfully elevated the total ACCase levels in plastids. The transformants displayed extended leaf longevity, a 2-fold increase in seed yield, and just about doubled the fatty acid production. Transplastomic tobacco plants expressing the exogenous *Delta-9* desaturase genes showed altered fatty acid profiles and an increase in their unsaturation level in both leaves and seeds (Craig et al. 2008). Plastid genetic engineering can also be efficiently used for synthesis of unusual fatty acids, such as very-long-chain polyunsaturated fatty acids (VLCPUFAs), which are generally absent from plant foods. As plastid engineering offers the advantage of engineering multiple genes in operons, it could allow the expression of four genes (three subunits ORF A, B, C of the polyketide synthase system, and the enzyme phosphopantetheinyl transferase), required for the production of VLCPUFAs (Rogalski and Carrer 2011).

## Biopolymer production

The production of biodegradable polymers as a substitute for petrochemical compounds through transgenic technology is a great challenge for plant biotechnologists (Neumann et al. 2005; Huhns et al. 2009). Polyhydroxybutyrate (PHB), which serves as a carbon storage molecule in the bacteria, has drawn considerable attention from industries due to its potential application in biodegradable plastics and elastic polymers. Polyhydroxybutyrate is synthesized from acetyl-coenzyme A, through the consecutive activity of three enzymes of bacterial origin: β-ketothiolase, acetoacetyl-CoA reductase, and PHB synthase. A number of genes encoding synthesis of biodegradable polyester have already been expressed in tobacco plastids with PHB expressions of approximately 0.006–0.1 % of dry weight of leaf samples (Lossl et al. 2003; Arai et al. 2004). Recently, Bohmert-Tatarev et al. (2011) reported the PHB expression up to 18.8 % dry weight of leaf tissue, with use of an optimized gene construct based on their similarity to the codon usage and GC content of the tobacco plastome. The plant-derived collagen and spider silk-elastin fusion proteins have immense uses in biomedical science (Scheller and Conrad 2005). Guda et al. (2000) successfully produced the bioelastic protein-based polymers by integration and expression of the biopolymer gene (EG121), in the tobacco plastid. However, its feasibility of production in quantities and at purities adequate for commercial spinning remains challenging. Recently, Xia et al. (2010) expressed spider dragline silk favorably in metabolically engineered *E. coli*, by overcoming the difficulties caused by its glycine-rich characteristics, thus providing new insight into optimal expression and synthesis of plastid-targeted silk proteins, possibly with increased yields and metabolic compartmentalization with minimal adverse effect on plant systems.

## Production of pharmaceuticals

Plastid transformation technology is set to become a major role player in the production of human therapeutic proteins. An increasing number of pharmaceutical proteins and vaccines have already been produced in the chloroplast of tobacco (Nugent and Joyce 2005; Daniell et al. 2009; Bock and Warzecha 2010; Gorantala et al. 2011; Lössl and Waheed 2011; Maliga and Bock 2011). In a number of cases, extraordinarily high level expression of foreign proteins was achieved in transplastomic tobacco. Abundant local expression of the human serum albumin (HSA) has been achieved in tobacco plastids as inclusion bodies, yielding a recovery of about 0.25 mg HSA/g fresh weight, which was well within the range of industrial-scale feasibility (Fernandez-San Millan et al. 2003). Oey et al. (2009) reported the maximum accumulation of a phage lytic protein, PlyGBS (>70 % of the TSP) and proved to be extremely stable in transgenic chloroplasts of tobacco. Genes encoding the human somatotropin (hST) (Staub et al. 2000), cholera toxin B subunit (CTB) (Daniell et al. 2001b), tetanus toxin C fragment (TetC) (Tregoning et al. 2003), anthrax protective antigen (PA) (Watson et al. 2004; Koya et al. 2005), HPV16 (human papillomavirus type 16), L1 antigen (Fernandez-San Millan et al. 2003; Lenzi et al. 2008), and antimicrobial peptides retrocyclin-101 (RC101) and protegrin-1 (PG1) (Lee et al. 2011) have been produced in transplastomic tobacco plants.

Production of two or more vaccine fusion proteins could possibly facilitate the easy purification and processing and reduction in the cost of production. Expression of foreign proteins in tobacco as fusion associates facilitated a significant accumulation of hST and interferon-gamma (IFN-g) (Daniell 2006); non-toxic CTB, genetically fused to 2L21(a linear antigenic peptide from the VP2 capsid protein of the canine parvovirus) (Molina and Veramendi 2009), and with human proinsulin (Ruhlman et al. 2007; Boyhan and Daniell 2011). Similarly, production of a multiepitope DPT vaccine fusion protein, containing immunoprotective exotoxin epitopes of *Corynebacterium diphtheriae*, *Bordetella pertussis*, and *Clostridium tetani*, has also been achieved in tobacco chloroplasts (Soria-Guerra et al. 2009). Gargano et al. (2005) reported the transplastomic potato plants with expression of the HPV16 L1 capsid protein fused to a His6 tag. An additional vaccine candidate, the E7 HPV type 16 oncoprotein fused with potato virus X coat protein (CP), was also expressed in tobacco chloroplasts (Morgenfeld et al. 2009). Recently, Waheed et al. (2011) developed a cost-effective alternative approach for VLP-based HPV vaccines. A modified *HPV-16L1* gene, which retained the ability to assemble L1 protein to capsomeres was expressed in tobacco chloroplasts. Capsomeres are considered relatively thermostable and are able to induce the immunogenicity to a level as that of VLPs (virus-like particles) (Schädlich et al. 2009; Lössl and Waheed 2011).

The chloroplast transformation system has also been explored for the production of HIV antigens (McCabe et al. 2008; Zhou et al. 2008). Transplastomic tobacco plants were able to accumulate human immunodeficiency virus type 1 (HIV-1) p24 (the major target of T-cell-mediated immune responses in HIV-positive individuals) protein up to about 2.5–4.5 % of the TSP with correct size and without any post-translational modifications, such as glycosylation or phosphorylation (McCabe et al. 2008). Zhou et al. (2008) successfully expressed the various HIV antigens (p24, Nef, p24-Nef, and Nef-p24) in tobacco and tomato plastids. Optimized *p24-Nef* fusion gene cassettes increased the p24-Nef antigen protein accumulation to approximately 40 % of the plant’s total protein. These results demonstrate the considerable potential of transgenic plastids to produce AIDS vaccine components at a low-cost and high yield.

Genetically engineered starch particles, designated as amylosomes (Dauvillee et al. 2010), were used to produce recombinant anti-malaria vaccines in the unicellular green algae, *C. reinhardtii*. Apical major antigen (AMA1) or major surface protein (MSP1), which is fused to the algal granule-bound starch synthase (GBSS), are efficiently expressed and bound to the polysaccharide matrix. The salient feature of this approach is that the starch is easy to purify and represents a protective environment for bound proteins, as GBSS is known to be remarkably stable with no detectable loss of activity, even after years of storage. This system should also be expedient to the production of any recombinant antigens, including vaccine candidates of viruses, bacteria, and other protozoan parasites, plus they could be deployed to starch-producing crop plants, including cereals and potatoes (Dauvillee et al. 2010). These results clearly indicate that plastid transformation is an effective plant-based production platform for next-generation vaccines.

## Limitations

The future of chloroplast genetic engineering for a wide range of applications gives us a reason to be optimistic. However, there are several areas of concern that will require attention if the full potential of this technology is to be realized. Low plastid transformation efficiencies of some of the species and inefficient gene expression in non-green plastids, such as potato tuber amyloplasts and chromoplast of tomato and pepper (Brosch et al. 2007; Kahlau and Bock 2008; Valkov et al. 2009), are major constraints for further extension of this technique in *Solanaceae*. Moreover, gene expression in non-green tissue plastids is largely uncharacterized, compared with leaf chloroplasts and the lack of appropriate tissue-specific regulatory sequences, which function in non-green plastids to achieve efficient transgene expression, is another major obstacle.

Although transgenes are generally efficiently targeted to their desired insertion site, unintended secondary homologous recombination events have been observed during plastid transformation that may hinder an efficient recovery of plastid transformants containing the desired transgene (Svab and Maliga 1993; Iamtham and Day 2000; Gray et al. 2009). It was suggested that these unwanted recombination events could be of common occurrence in chloroplast transformation experiments, as UTRs for plastid transgenes are usually derived from endogenous chloroplast genes (Rogalski et al. 2006). Ahlert et al. (2003) reported that unwanted recombination events were avoided between the *psbA*-derived 3′ end of the chimeric *aadA* gene and the endogenous *psbA* locus using a short version of the *psbA* 3′ end. Similarly, the length of the 5′ UTR was also reduced to minimize the probability of an unwanted homologous recombination in plastid transformants (Maliga and Bock 2011). Thus, the use of truncated UTRs, and keeping a low number of UTRs is a highly desirable and efficient approach for vector construction. Nevertheless, it is important for the interpretation of RFLP analyses, which are commonly conducted to demonstrate transgene integration and homoplasmy of transplastomic plants (Rogalski et al. 2006).

Most plastid genes are part of operons, expressed as polycistronic mRNAs, and these mRNA transcripts are post-transcriptionally processed into mono- or oligo-cistronic units, presumably by specific endonucleolytic cleavage (Herrin and Nickelsen 2004; Zhou et al. 2007). Although several previous studies have suggested that the expression of transgenes from polycistronic mRNAs is possible (Staub and Maliga 1995; Quesada-Vargas et al. 2005), poor translation of polycistronic mRNAs has been presumed to be responsible for the cases where transgene expression was drastically low (Nakashita et al. 2001) or unsuccessful altogether (Magee et al. 2004). In most of the cases, this approach has failed due to fundamental differences in operon expression between bacteria and plastids. To overcome this problem, Zhou et al. (2007) described the use of small intercistronic expression elements (IEE), capable of generating stable translatable monocistronic mRNAs through intercistronic cleavage. Separation of transgenes by IEE promotes transgene stacking in operons, thus expanding the range of applications of transplastomic technology (Zhou et al. 2007). Applications of the IEE can be extended to the introduction of multiple disease resistance, the co-expression of selectable marker and reporter genes, as well as the engineering of complex biochemical pathways (Ye et al. 2001), and the production of biopharmaceuticals in plastids (Bock 2007).

Highly variable expression of foreign proteins in tobacco chloroplasts has been reported, ranging from 0.002 to 72 % of the TSP (Lee et al. 2006; Oey et al. 2009; Sim et al. 2009; Ruhlman et al. 2010). Nevertheless, massive expression of foreign proteins resulted in phenotypic alterations and delayed plant development in transplastomic plants (Chakrabarti et al. 2006; Oey et al. 2009) due to severe exhaustion of the endogenous gene expression of the chloroplast evident from the strongly downregulated RuBisCO, which constitutes the major leaf amino acid store (Oey et al. 2009; Bally et al. 2009). The constitutive expression of biopolymers and metabolic pathway enzymes in plastids also resulted in mutant phenotypes, adversely affecting the growth and development of transplastomic plants because of either metabolite toxicities, interference with photosynthesis, or disturbance of the plastid endomembrane system (Lossl et al. 2003; Neumann et al. 2005; Hennig et al. 2007; Huhns et al. 2009).

Protein stability has recently emerged as a major bottleneck to foreign protein accumulation in transgenic plastids (Apel et al. 2010; Elghabi et al. 2011b). Although several recombinant proteins were expressed to levels more than 10 % of the TSP (reviewed in Daniell et al. 2009; Bock and Warzecha 2010), there are several cases where a very low accumulation of foreign proteins was reported (Birch-Machin et al. 2004; Bellucci et al. 2005; Lee et al. 2006). It appears that accumulation of foreign proteins in transgenic chloroplasts is often limited by protein stability (Birch-Machin et al. 2004; Zhou et al. 2008; Oey et al. 2009), although lack of RNA stability can also be responsible for unsuccessful expression of plastid transgenes (Wurbs et al. 2007; Elghabi et al. 2011b). Apel et al. (2010) demonstrated that major protein stability determinants are located in the N terminus, and the penultimate N-terminal amino acid residue has an important role in determining the protein half-life. Recently, Elghabi et al. (2011b) also investigated the possibility of enhancing the expression of an unstable recombinant protein, the HIV-1 fusion inhibitor cyanovirin-N (CV-N) in transgenic plastids by protecting its N and/or C-terminus with polypeptide sequences taken from the highly stable proteins, GFP and PlyGBS. It is possibly by impeding the endoribonucleolytic cleavage of the CV-N coding region and thus exerts the observed stabilizing effect on the mRNA (Elghabi et al. 2011b). In similar experiments, an efficient fusion of a downstream box, composed of the 10–15 codons immediately downstream of the start codon, allowed high-level accumulation of active bacterial β-glucosidase in tobacco chloroplasts (Gray et al. 2011). Significant increase in transcript stability can be achieved by inserting sequences from stable mRNAs between the 5′ UTR and the coding region of the transgene of interest and highlights a possible solution in all those cases, where transgene expression is limited by mRNA accumulation (Elghabi et al. 2011b).

In order to confirm the homoplasmy of all transplastomic lines, it is necessary to perform seed assays, which are the most sensitive tests available to assess homoplasmy. A lack of segregation of antibiotic resistance in the T1 generation demonstrates the homoplasmy and confirms the uniparental maternal transgene inheritance (Hagemann 2002; Maliga 2004). However, obtaining homoplasmic transplastomics is quite challenging, and several additional rounds of selection adversely affect the regeneration efficiency of recalcitrant species. Even after several rounds of antibiotic selection, complete intraorganellar homoplasmy is difficult to achieve in species with poor regeneration capacity (Bock 2001) and upon transfer to the selection-free media, segregation of plastids with wild-type plastid genomes reoccurs, thereby resulting in the heteroplasmy of transplastomics (Wei et al. 2011).

Transgene containment is another key concern in genetically modified crops, especially for those species with outcrossing wild relatives. Consequently, engineering foreign genes in the chloroplast genome would provide enhanced transgene containment by virtue of uniparental maternal inheritance of plastids. However, there are two mechanisms by which plastid transgenes can escape through pollen at a low frequency: occasional paternal/biparental transmission of plastids (Ruf et al. 2007; Svab and Maliga 2007; Matsushima et al. 2008) and transfer of transplastome genes to the nuclear genome (Huang et al. 2003; Stegemann et al. 2003; Sheppard et al. 2011). Transgene containment, via maternal inheritance, would not be applicable to a few crops, such as alfalfa and evening-primrose, in which maternal inheritance is not a rule. They exhibit an equal distribution of plastids during the first pollen mitosis into the generative and vegetative cells; therefore, sperm cells transmitting plastids into egg cells during fertilization, is called biparental plastid transmission (Daniell 2007; Matsushima et al. 2008). In tobacco, a very low frequency of occasional paternal transmission of transgenic plastids was observed under experimental conditions in the range of 1 × 10^–4^ to 2.86 × 10^–6^ (Ruf et al. 2007; Svab and Maliga 2007). The frequency of occasional paternal transmission of transgenic plastids under field conditions, where transgenic and non-transgenic plants were grown separately, suggested being in the range of 10^−8^ (Ruf et al. 2007), thereby demonstrating that plastid transgene transmission is less likely to occur under field conditions. Although organellar DNA transfers very frequently into the nucleus, most of it is quickly deleted, decayed, or is alternatively scrapped, and a very small proportion of it gives rise, immediately or eventually, to functional genes (Lloyd and Timmis 2011). Nuclear transfer of the plastid gene would normally not result in transgene expression, due to the absence of a nuclear promoter, yet accidental integration and subsequent rearrangements could bring a transgene into context with an existing nuclear promoter (Stegemann and Bock 2006; Lloyd and Timmis 2011).

Although the level of containment conferred by the transplastomic plants is suggested to be convincingly sufficient (Ruf et al. 2007; Svab and Maliga 2007), there are numerous applications where the absolute containment for such transplastomic lines is required, for instance, in the case of production of industrially important therapeutic proteins and vaccines. To achieve this, plastid transformation should be accompanied with one or more viable containment strategies, such as reversible cytoplasmic male sterility (Ruiz and Daniell 2005), genetic use restriction technology, and transgene mitigation strategies (Ruf et al. 2007; Day and Goldschmidt-Clermont 2011). Another approach, which might help to minimize functional transgene escape through pollen, is the incorporation of RNA editing sites in the plastid transgene. Sheppard et al. (2011) reported that the RNA editing of chloroplast transgenes may reduce the functional gene transfer to nucleus but may not eliminate the plastid-to-nucleus gene transfer. An even more efficient approach that has been employed in the past to avoid functional gene transfer to the nucleus is the use of chloroplast group II introns. Chloroplast group II introns are known to interrupt reading frames in fungal and plant mitochondria, in plastids, and in bacteria. Bock and Maliga (1995) investigated the splicing of *atpF*, a plastid group II intron. They observed that after the insertion of *atpF* intron into a chimeric plastid *uidA* gene, the *GUS* reporter gene expression became dependent on the correct splicing of intron. Recently, in a similar report, Petersen et al. (2011) investigated the putative role of two group II introns that interrupt a plastid gene (*ycf3*), whose removal is essential for synthesis of a functional ycf3 polypeptide and thus for photosynthetic activity. Based on their results, they suggested that the splicing of one intron can depend on the presence of another intron, and the group II introns can have a selective value in that their loss can cause a decline in fitness (Petersen et al. 2011). To date, no single strategy has been established that is broadly applicable, but a combination of approaches would be most efficient for engineering eco-friendly transgenic crops.

A major concern with plastid genetic engineering of higher plants might be the use of antibiotic-resistance genes as the selectable markers. The large number of chloroplast genome copies in a plant cell as well as the prokaryotic features of their gene expression machinery might enhance the probability of horizontal gene transfer from plants to bacteria living in the soil or a gastrointestinal tract. However, to date, such risks have been found to be negligible. Moreover, the spread of antibiotic-resistance genes from crops among bacteria does not signify any selective advantage because various resistance genes and other genetic determinants are already naturally present in the environment (Demanèche et al. 2008; Talianova and Janousek 2011). Nevertheless, the removal of antibiotic marker genes and, following alternative strategies, possibly avoids such risks associated with transplastomic plants. The continuous monitoring and evaluation of potential risks associated with transgenic plants would further ensure the biosafety and ease the public concerns of GM crops.

Finally, the financial and political obstacles that hinder the introduction of new vaccines in developing countries are major challenges of these research programs. Design of translational research programs is still in its infancy, and it is important to design them in a way that is responsive to the needs of national policy makers of any particular country.

## Future perspectives

Several improvements in plastid transformation vectors, transformation procedures, selection systems, and regeneration protocols have recently made it possible to produce industrially important proteins, plus plants with important agronomic traits to a somewhat greater extent. Plastid genetic engineering can be effectively used to modulate entire metabolic pathways or even to induce the expression of pathways in organs where they do not normally occur. The development of non-green plastid expression vectors remains an important goal for the realization of many of the potential benefits of plastid genetic engineering. Generally, potato amyloplasts exhibit a low transcription rate and increased transcript stability of plastid genes (Brosch et al. 2007; Valkov et al. 2009) and foreign protein accumulation was found to be several-fold lower in non-photosynthetic microtubers than in green leaves (Sidorov et al. 1999). Although transplastomic tomato leaf chloroplasts accumulated high level of foreign proteins (>40 % of the TSP), fruit chromoplasts were able to express the transgene to about 50 % of the expression levels achieved in leaf chloroplasts (Ruf et al. 2001). Expression levels achieved in potato amyloplasts and tomato chromoplasts (Sidorov et al. 1999; Zhou et al. 2008; Valkov et al. 2011) may be adequate to manipulate the expression of enzymatic proteins for metabolic engineering purposes but are still too low to exploit as a production platform for proteins of pharmaceutical or industrial interest. Recently, a systematic characterization of gene expression in tuber amyloplasts and chromoplasts of tomato and pepper revealed that gene expression in such organelles is generally impaired, with multistep control occurring at transcriptional, post-transcriptional, and translational levels (Brosch et al. 2007; Kahlau and Bock 2008; Valkov et al. 2009). However, some transcripts, such as the transcript of the fatty acid biosynthesis gene, *accD*, displayed relatively high gene expression activity in potato tubers and in tomato and pepper fruits. All these studies have allowed the tentative identification of candidate regulatory sequences, which could potentially improve transgene expression in nongreen plastids (Kahlau and Bock 2008; Valkov et al. 2011).

Plastid genetic engineering has been extended to edible vaccine production, which could minimize the downstream processing costs and the risks associated with conventional vaccine production systems. In addition to added stability, correct disulfide bond formation in some therapeutic proteins is an absolute requirement for functional, biologically active molecules (Ludwig et al. 1985; Dertzbaugh and Cox 1998). Plastid genetic engineering would be an ideal system for the synthesis of sulfur-rich storage proteins, as protein disulfide isomerase, the enzyme responsible for the formation and breakage of disulfide bonds between cysteine residues within proteins, is known to be active in plastids (Kim and Mayfield 2002; Alergand et al. 2006). Another interesting feature of chloroplast transformation is the absence of a glycosylation pathway (Fernandez-San Millan et al. 2003; McCabe et al. 2008), which provides a unique opportunity to express therapeutic proteins free of glycosylation. Weeda et al. (2009) described the role of potato multicystatin (a multidomain Cys-type protease inhibitor), which facilitates the high accumulation of proteins in developing tubers and prevents the premature proteolysis of storage proteins in fully developed tubers by inhibiting Cys-type proteases (Weeda et al. 2009). These results could possibly provide a new opportunity to increase the foreign protein accumulation and storage in potato tubers.

Oral delivery of vaccines, expressed in plant cells would reduce the costs associated with purification, processing, cold storage, transportation, and delivery, and would be more efficacious than injectable vaccines (Arlen et al. 2008). To date, most of the chloroplast-based vaccines have been produced in tobacco, but tobacco is not suitable for oral delivery of vaccines. Tomato and sweet peppers are two important *Solanaceae* crops, which are consumed as raw vegetables and have enormous scope for edible vaccine production. Green tomatoes accumulated the p24-Nef fusion protein to approximately 2.5 % of the TSP; however, in red ripe tomatoes, expression of p24-Nef protein was hardly detectable when compared with leaves, thereby being similar to the situation in older tobacco and tomato leaves (Zhou et al. 2008). This is because most plastid genome-encoded genes involved in photosynthesis are downregulated in non-photosynthetic tissues (Kahlau and Bock 2008). Chloroplast transformation of stay-green tomato/capsicum phenotypes for vaccine production would provide a solution for this. Alternatively, chloroplast transformation coupled with downregulation of “*stay-green*” (*SGR*) genes, which are involved in regulation of plant senescence (Roca et al. 2006; Barry et al. 2008) would possibly facilitate the increased accumulation of vaccine peptides through delaying the fruit ripening or by allowing the coexistence of the chromoplasts and photosynthetically active chloroplast in the ripened fruits.

The use of inducible promoter systems, which trigger transgene expression upon induction by chemical or physical means, would avoid the deleterious effects caused by constitutive expression of transgenes in chloroplasts and would further ensure the security and control of production in GM plants. A nuclear-encoded ethanol-inducible plastid-targeted T7 RNA polymerase, which transcribes plastid transgenes from a T7 promoter system, has already been used for inducible expression of PHB in transplastomic tobacco (Lossl et al. 2005). A recently identified synthetic riboswitch, which functions as an efficient translational regulator of gene expression in plastids (Verhounig et al. 2010), could provide a novel tool for plastid genome engineering in the near future, as it facilitates the tightly regulated inducible expression of transgenes. Genomic approaches are being used to compare the expression of genes in chloroplasts, amyloplasts, and chromoplasts, with the aim of identifying regulatory sequences, which support high gene expression in non-green plastids. Candidate regulatory sequences that can potentially improve plastid (trans) gene expression in amyloplasts have already been identified (Valkov et al. 2009). Attempts were also made to improve the transformation efficiency, with the use of novel vectors containing species-specific flanking sequences for homologous recombination in the large single copy (LSC) region of the plastome (Scotti et al. 2011; Valkov et al. 2011). Experiments with vectors containing different promoters and terminators as well as the use of species-specific flanking and regulatory sequences have already been seen as promising. Further efforts should be made to develop tuber- and fruit-specific plastid expression vectors for plastid transformation in non-green plant organs.

## Conclusion

Considerable progress has been made in understanding the plastid transgene expression and in unraveling the potential of the technology for future biotechnological applications (Bock 2007; Verma et al. 2008; Bock and Warzecha 2010; Maliga and Bock 2011). A greater extent of foreign protein accumulation was achieved in transplastomic tobacco plants. A significant improvement in plastid transformation efficiency is likely to be of considerable value for future implementation in other *Solanaceae* crops. An efficient shoot regeneration system is the key to successful production of transplastomic plants, and with improved regeneration methods and genotypes with a high regeneration capacity, a further higher frequency of transplastomic plants could be achieved. Production of marker-free transplastomic plants would ease public concern and increase consumer preferences. Besides maximizing the expression of foreign proteins, careful optimization of the expression level is also required in order to minimize the adverse effects on plant system. The widespread use of plastid genetic engineering is still a long way off, especially in the case of expression of transgenes in non-green plastids. However, with the recent advances in gene expression technologies and plastid genomic studies, expression of the foreign in non-green organ plastids would be quite feasible in the near future.
